# Functional region prediction with a set of appropriate homologous sequences-an index for sequence selection by integrating structure and sequence information with spatial statistics

**DOI:** 10.1186/1472-6807-12-11

**Published:** 2012-05-29

**Authors:** Wataru Nemoto, Hiroyuki Toh

**Affiliations:** 1 Computational Biology Research Center (CBRC), Advanced Industrial Science and Technology (AIST), AIST Tokyo Waterfront Bio-IT Research Building. 2-4-7 Aomi, Koto-ku, Tokyo 135-0064, Japan; 2 Division of Life Science and Engineering, School of Science and Engineering, Tokyo Denki University (TDU), Ishizaka, Hatoyama-cho, Hiki-gun, Saitama, 350-0394, Japan

## Abstract

**Background:**

The detection of conserved residue clusters on a protein structure is one of the effective strategies for the prediction of functional protein regions. Various methods, such as Evolutionary Trace, have been developed based on this strategy. In such approaches, the conserved residues are identified through comparisons of homologous amino acid sequences. Therefore, the selection of homologous sequences is a critical step. It is empirically known that a certain degree of sequence divergence in the set of homologous sequences is required for the identification of conserved residues. However, the development of a method to select homologous sequences appropriate for the identification of conserved residues has not been sufficiently addressed. An objective and general method to select appropriate homologous sequences is desired for the efficient prediction of functional regions.

**Results:**

We have developed a novel index to select the sequences appropriate for the identification of conserved residues, and implemented the index within our method to predict the functional regions of a protein. The implementation of the index improved the performance of the functional region prediction. The index represents the degree of conserved residue clustering on the tertiary structure of the protein. For this purpose, the structure and sequence information were integrated within the index by the application of spatial statistics. Spatial statistics is a field of statistics in which not only the attributes but also the geometrical coordinates of the data are considered simultaneously. Higher degrees of clustering generate larger index scores. We adopted the set of homologous sequences with the highest index score, under the assumption that the best prediction accuracy is obtained when the degree of clustering is the maximum. The set of sequences selected by the index led to higher functional region prediction performance than the sets of sequences selected by other sequence-based methods.

**Conclusions:**

Appropriate homologous sequences are selected automatically and objectively by the index. Such sequence selection improved the performance of functional region prediction. As far as we know, this is the first approach in which spatial statistics have been applied to protein analyses. Such integration of structure and sequence information would be useful for other bioinformatics problems.

## Background

Many methods have been developed to predict the functional regions of a protein [1]. One of the most effective strategies is the detection of conserved residue clusters on the tertiary structure of the protein [2-9]. Various methods, such as Evolutionary Trace [2], PatchFinder [10,11] and ConSurf [5], have been developed based on this strategy. In such methods, at first, the homologous amino acid sequences of a prediction target are collected, and a multiple sequence alignment (MSA) of the sequences is constructed. Then, the conserved residues are identified among all sites in the MSA, which are assigned to the corresponding residues on the tertiary structure of the protein. Finally, the clusters of conserved residues on the structure are predicted as the functional regions. In such approaches, a problem has remained; that is, how to select the appropriate homologous sequences for the identification of conserved residues. It is empirically known that a certain degree of sequence divergence in an MSA is essential for the identification. However, there is no concrete criterion for the sequence divergence that is generally applicable to all cases [12]. The selection of the sequences for the MSA to identify conserved residues is thus unavoidably subjective. In addition, the prediction performance strongly depends on the sequence divergence [12]. Therefore, an objective criterion for the divergence is required to achieve high functional region prediction performance.

Homologous sequences with exactly the same function are considered to be appropriate for functional region prediction. The difficulty in the selection of such sequences resides in the fact that homologous proteins do not always share the same function, although they are derived from a common ancestral protein. For example, gene duplication is considered to be an evolutionary mechanism for a protein to acquire a new function. An additional copy generated by gene duplication allows one of the genes to accept mutations, which cannot be tolerated by a single copy gene. Such drastic mutations often lead to the functional divergence of the duplicated genes. We can see such an example in the relationship between C-type lysozyme and α-lactalbumin, which both belong to the C-type lysozyme family [13]. C-type lysozyme attacks peptidoglycans and hydrolyzes the glycosidic bond that connects N-acetylmuramic acid with the fourth carbon atom of N-acetylglucosamine [14]. α-lactalbumin is a non-enzymatic homologue of C-type lysozyme, and acts as the regulatory component of the lactose synthase enzyme system [13]. The catalytic sites and substrate binding sites for the lysozymes are substituted with different amino acids in α-lactalbumins, although the two proteins share about 40% sequence identity. Consider the situation in which we want to predict the functional regions of lysozyme. Then, the MSA of the lysozyme sequences is required. However, if the MSA includes the α-lactalbumins in addition to the lysozymes, then the accurate prediction of the catalytic residues of the lysozymes is difficult, based on the residue conservation. In such a case, we should exclude the sequences of α-lactalbumin, and use only those of the C-type lysozyme for the identification of conserved residues. This example highlights the difficulty in selecting the appropriate sequences with the same functions and using them for functional region prediction.

Roughly speaking, there are two ways to select homologous sequences with the same function for functional region prediction. One simple way to select homologous sequences is the selection based on their sequence identities to the prediction target [1]. However, there is a problem with this method. Todd *et al*. investigated the conservation of molecular functions by examining the EC numbers of the homologous enzymes. In most cases, the first three digits of the EC numbers are the same when the sequence identity between any two proteins is greater than 40% [15]. Even if the sequence identity is around 30%, 95–90% of homologous enzymes share the first three digits of the EC numbers. If the sequence identity is less than 30%, then the conservation in the EC number is degraded [15]. The observations suggest that more than 30% or 40% sequence identity may be used as an empirical criterion to construct the appropriate set of homologous sequences for functional region prediction. In contrast, Rost *et al*. [16] reported that less than 30% of homologous enzyme pairs share the same EC number, even when the sequence identity is greater than 50%. Thus, the relationship between the conservation in the EC number and the sequence identity is still debatable. Sequence identity-based selection is not always appropriate for the selection of sequences with the same function. Another way is to select the closely related sequences with the annotations, such as a protein name and its biochemical functions. However, this selection method also has some limitations. Firstly, some hypothetical proteins are annotated based only on the sequence identity or similarity with the closely related homologous proteins. Secondly, many sequences are simply described as ‘hypothetical protein’ or ‘function unknown’, even when the retrieved sequences from the databases display significant sequence similarity to the protein under consideration [1]. Therefore, the sequence selection methods that rely on the percent sequence identity to the query are insufficient for functional region predictions. To address this problem, two methods have been developed.

One of the methods was reported by Aloy *et al. *[8]. They developed an automatic method to predict the functional regions of a protein by using sequence and structure information. In their method, the clustering of the conserved residues on the tertiary structure is evaluated. If no cluster is identified, then the MSA is reconstructed by removing the distant homologues to the target protein, according to the evolutionary relationships suggested by a phylogenetic tree. The process is iterated until the cluster of conserved residues is identified.

Recently, another approach employing structure and sequence information was proposed by Mihalek *et al.*[12,17]. They used a “residue clustering measure” to indicate the appropriateness of a set of sequences for functional region predictions [12,17]. The measure quantifies the degree of clustering of the evolutionarily important residues in the tertiary structure of a protein. In addition, the measure attaches greater importance to the clustering of the residues that are far from each other on the primary structure. The set of sequences selected by the measure led to better performance of their functional region prediction by the real valued Evolutionary Trace (rvET)-based method [18].

We addressed a similar problem to that studied in Mihalek’s work by a different approach. We developed an index to quantify the appropriateness of a set of homologous sequences, which is implemented in the method to predict the functional regions of a protein. Structure and sequence information were integrated by spatial statistics into the index, which represents the degree of clustering of the conserved residues on the tertiary structure of the protein. Spatial statistics is a large branch of statistics that considers both the spatial positions and attributes of the data, and analyzes the spatial relationships among the data [19]. The performance of functional region prediction, using the set of sequences selected by the index, was better than that using the set selected by percent sequence identity. The advantage of our method is the automatic and objective construction of an appropriate sequence set for functional region prediction. The virtues and pitfalls of our method will be discussed.

## Methods

At first, we will describe the datasets used for the performance evaluation of our method. We will then explain the prediction procedure of our method, and describe the evaluation methods.

### Datasets

In this work, we predicted the functional regions of monomeric enzymes with structures available in the Protein Data Bank (PDB), and evaluated the prediction accuracy. Structure, sequence and catalytic site data were required for the prediction and evaluation. Data sources and manipulation methods are described below.

#### Structure data

We used monomeric enzymes for the performance evaluation of our method. At first, we selected the enzymes with catalytic sites listed in the Catalytic Site Atlas [20]. Second, we adopted monomeric enzymes, if the complex state of the protein is registered as monomeric in three databases, ProtBuD [21], 3DComplex.org [20], and PQS [20]. The non-redundant target protein set was constructed by adopting only a representative protein from the clusters defined in 3DComplex.org, where the structures are hierarchically classified by similarities in the quaternary complex state, the tertiary structure and the primary sequence. We used the file corresponding to “QS30 level” for the hierarchical classification, where two protein complexes are clustered if the complex topologies are identical, the structures are similar (based on SCOP), and the percent sequence identity between the two protein sequences is larger than 30%. In addition, if the sequence of a structure has at least 10 homologous sequences with more than 60% sequence identity, then the structure is used as a target protein for the performance evaluation of our method. Otherwise, the protein was not used as a target. Considering the report by Rost *et al*. [16], the proteins with about 50% identity to the target may not share the same function. Therefore, we used a slightly more rigorous criterion, 60%, for the selection of test sets. The number of analyzed PDB chains was 54. All PDB IDs and Chains are listed in the Supporting Information.

#### Sequence data

The sequence corresponding to the target structure and its homologous sequences were collected from the RefSeq database [22] by BLAST [23].

#### Catalytic site information

Catalytic site information was obtained from the Catalytic Site Atlas [24], which contains two information sources. One is the literature, where catalytic sites are confirmed experimentally. The other is the detection of catalytic sites based on a BLAST sequence search [23]. We utilized both sources.

### Prediction procedure

The key idea of our method is the integration of structure and sequence information within the prediction procedure, by the application of spatial statistics. At first, we will describe the underpinning of our method, and then we will provide a detailed explanation of the prediction procedure.

### Underpinning of our method

#### Data representation of a protein structure as a residue-residue interaction network

A protein structure is represented as an undirected graph *G* (*N*, *E*). *N* indicates a set of nodes. Each node corresponds to a constituent residue of the protein structure. *E* indicates a set of edges, each connecting a pair of contact residues. In order to construct a graph representation, at first, the Voronoi areas are automatically generated on the molecular surface of the protein by PROVAT [25], which is software that enables the computation and visualization of Voronoi tessellations of proteins and protein complexes. In the tessellation process, we defined C_β_ as the representative atom for all types of amino acids, except for Gly, for which C_α_ is the representative atom. Second, two contacting residues are defined as the residues with corresponding Voronoi areas adjacent to each other, and the undirected graph is generated.

#### Assumption of functional region prediction

Two assumptions for functional regions of proteins were adopted in this study. Firstly, we assumed that most of the functional regions of proteins are exposed or at least semi-buried on the molecular surface. Bartlett *et al*. [26] reported that most of the catalytic residues have low relative solvent accessibility. Among them, however, the ratio of catalytic residues with 0% accessibility is only 5%. That is, most of the catalytic residues are located at positions that are somewhat solvent accessible. Actually, several prediction and theoretical studies have found that the functional regions tend to be located on the molecular surfaces of protein structures [2,4,5,27-29]. Therefore, we focused only on the surface residues of each monomer in this study. Secondly, we assumed that the amino acid residues conserved among homologues are abundant at the functional regions, due to functional constraints. Based on the two assumptions, we have developed a method to predict the functional regions of a protein, by examining the clustering of conserved residues on the protein surface with spatial statistics techniques.

#### Basis of the application of spatial statistics to protein analysis

The basis of our strategy is the theory of spatial statistics, in which not only the attributes but also their spatial coordinates are utilized. This enables us to evaluate the spatial relationship of the attributes. This data structure is used in epidemiology, geostatistics and geometric information system research [19]. Many statistical techniques for spatial data analysis have been developed in these fields [19]. Among them, some techniques developed by Moran and his colleagues are often used to investigate the global distribution of the attributes, and to detect local clusters [30-32]. Spatial autocorrelation is a measure to deal simultaneously with similarities in the locations of spatial objects and their attributes [19]. If the spatial objects with similar locations also share similar attributes, then the distribution pattern of the attributes in space as a whole is said to exhibit positive spatial autocorrelation. Conversely, negative spatial autocorrelation exists when the nodes that are close together in space tend to have more dissimilar attributes than those that are farther apart. Zero spatial autocorrelation occurs when the attributes are independent of the location. There are two approaches to analyze the spatial autocorrelation of spatial data, which differ from each other in terms of the analytical resolution [19]. In the first approach, the global features of the spatial distribution of attributes are explored. The Moran scatterplot is used for the exploratory analysis to visualize the global distribution pattern of the attributes. In the Moran scatterplot, the average of the attributes of neighboring nodes is calculated as follows:

(A) Averageofattributesofneighboringnodesofnodei=∑j=1,j≠iNwijAttributej∑j=1,j≠inwijwij={0:dij>D(Å)1:dij≤D(Å)

and is plotted as a function of the score of node *i*. Thus, the slope of the Moran scatterplot indicates the degree of spatial autocorrelation (DSPAC). The second approach is the detection of the local spatial factors that cause the spatial autocorrelation [19]. Local Moran’s I is a score to detect local clusters of nodes with high or low attributes. The mathematical details of the score will be outlined later in the section that describes the flow of our prediction method.

In our work, we applied Moran scatterplot [33] and Local Moran’s I [30,31] to predict functional regions by using the C_β_ atom of a surface residue, the conservation score and the residue contact as the node, the attribute and the edge, respectively. In a Moran scatterplot for a protein, the average conservation scores of neighboring residues are plotted as a function of the conservation score of a residue. Therefore, we can expect to observe a positive spatial autocorrelation in the Moran scatterplot where conserved residues are spatially surrounded by conserved residues, and where unconserved residues are surrounded by unconserved residues. The slope of the Moran scatterplot, in other words, DSPAC, indicates the strength of the contrast. We adopted DSPAC as an index of the appropriateness of a set of homologous sequences for functional region prediction. More precisely, we generated a set of homologous sequences of a target protein, and divided the set into some subsets. Among the subsets, we chose the subset with the maximum DSPAC. Further details of the application are described below.

#### Spatial autocorrelation for the conservation scores of protein residues

In this section, we describe the analyses of the spatial autocorrelation from two different viewpoints.

#### Spatial autocorrelation for the conservation scores of the residues on the molecular surface of a protein

**Figure 1:**
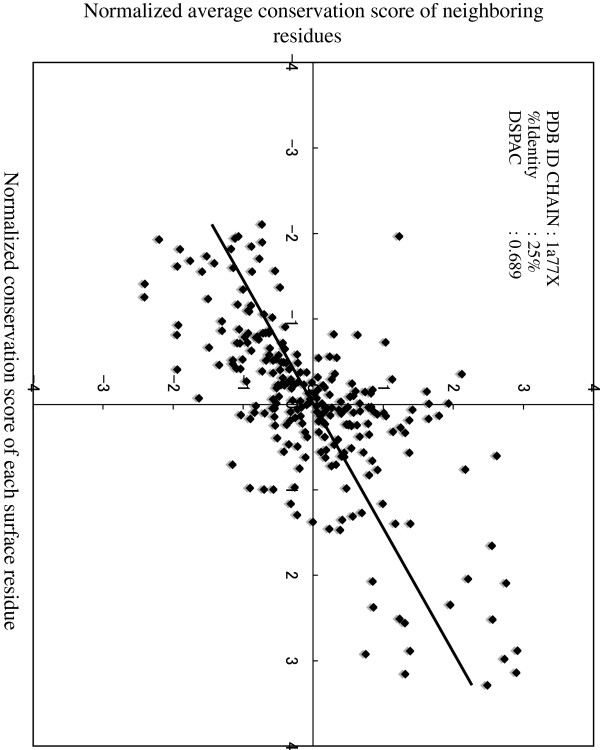
**Moran scatterplot of the conservation scores of the residues on the molecular surface of flap endonuclease-1 (1A77).** The averages of the normalized conservation scores of neighboring residues are plotted as a function of the normalized conservation score of each surface residue.

We generated a Moran scatterplot for the conservation scores on the surface of flap endonuclease-1 (1A77 chain A) [34], as an example (Figure 1). The conservation score was calculated at each alignment site by the following method [35]:

(B) $$\begin{array}{cc} \text{Cons}\left(i\right)=\frac{\sum_{j}^{N}\sum_{k>j}^{N}W_{j}W_{k}\text{Mut}\left(s_{j}(i),s_{k}(i)\right)}{\sum_{j}^{N}\sum_{k>j}^{N}W_{j}W_{k}} & \\ \text{Mut}\left(a,b\right)=\left\{ \begin{array}{c} \frac{m(a,b)-\text{min}\;\left(m\right)}{\text{max}(m)-\text{min}\;\left(m\right)}, \end{array}\begin{array}{ll} \text{if} & a\neq \text{gap}\;\text{and}\\ & b\neq \text{gap}\;\\ & 0,\text{otherwise} \end{array}\right. \end{array}$$

where *m*(*a, b*) is the BLOSUM62 [36] score between residues *a* and *b*. *max*(*m*) and *min*(*m*) are the maximum and minimum values among the scores, respectively. *W*_*j*_ is a weighting factor for sequence *j* to reduce the taxonomic bias among the set of sequences, which is calculated using the Henikoff-Henikoff weighting factor method [37]. *Cons* (*i*) ranges from 0.0 to 1.0. Values of *Cons* (*i*) closer to 1.0 reflect more conservation of the alignment site *i*. In this analysis, the sequences with *X*(%) or greater sequence identities to the sequence of a target protein were collected, to calculate the conservation score. The neighboring residues of residue *i* are defined as follows. Firstly, as described above, we defined the surface residues by PROVAT [25]. Second, the surface residues with C_β_ within *D*(Å) from that of surface residue *i* were adopted. Hereafter, this *D* is referred to as the neighboring residue threshold. Finally, in order to remove the residues that are close in direct distance but distant on the molecular surface, the residues with separation degrees from residue *i* that are less than or equal to 3 were selected. The pair of *X* and *D* corresponding to the maximum DSPAC was chosen as the thresholds. *X* was shifted from 85% to 10%, with intervals of 5%. *D* was shifted from 5.0Å to 9.0Å, with intervals of 0.5Å. In the analysis of flap endonuclease-1, the maximum DSPAC was 0.689, with 25% for *X* and 9.0Å for *D*.

In the same way, we calculated the DSPACs of 54 proteins in the dataset. The average DSPAC for the dataset is 0.741 (σ = 0.078). Next, we performed a test of no correlation for each DSPAC. Null hypotheses about all proteins, for which there is no correlation between the conservation score of residue *i* and the average of the conservation scores of the residues neighboring residue *i,* were rejected at the significance level of 0.05. Thus, we observed spatial autocorrelation for the conservation scores; that is, there is a tendency for conserved residues to be surrounded by conserved residues on the surface of the structure, while unconserved residues are surrounded by unconserved residues.

#### The selection of an appropriate set of homologous sequences for functional region prediction

We will now explain our strategy to automatically select an appropriate set of homologous sequences for functional region prediction. As described above, we used the DSPAC as an index to evaluate the appropriateness of a set of sequences for functional region prediction. The DSPAC is estimated by calculating the correlation coefficient of a Moran scatterplot about the conservation scores of a protein, which corresponds to the slope of the regression line in the Moran scatterplot. For this purpose, we constructed a set of homologous sequences of a target protein, and divided the set into some subsets. We chose the subset with the maximum DSPAC. Details are described below with a model case. Hereafter, suppose that the neighboring residue threshold, *D,* corresponding to the maximum DSPAC is known in advance for simple explanation.

**Figure 2:**
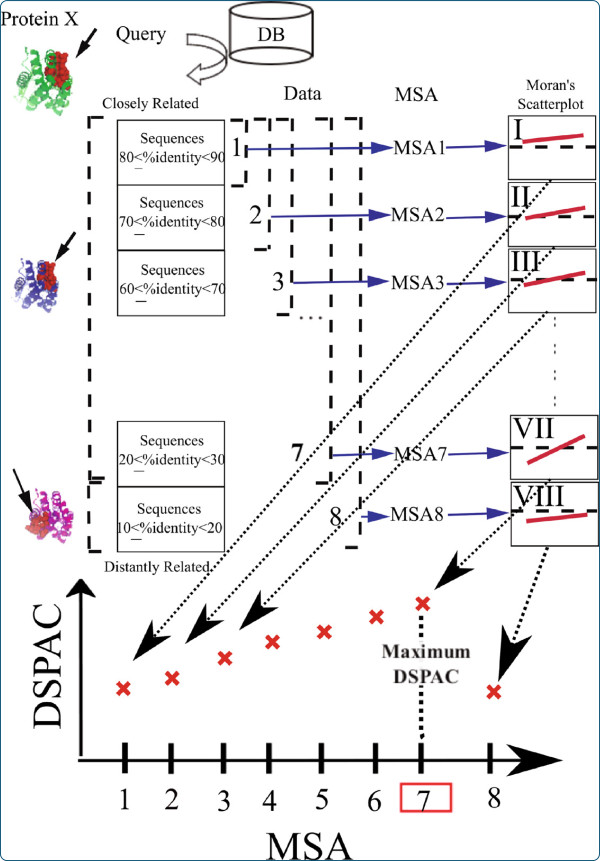
Schematic illustration of the DSPAC-based sequence selection.

Suppose that we have a list of 80 homologous sequences of an imaginary Protein X, for which the crystal structure is available (Figure 2). All sequences are retrieved by a sequence search, using Protein X as a query. The retrieved sequences are arranged in descending order of the percent sequence identity to Protein X. Closely-related homologous sequences of Protein X are near the top of the list. In contrast, distantly-related homologous sequences are near the bottom. Then, eight distinct datasets (DATA*i* : *i* = 1 ~ 8) are constructed, by selecting 10 × *i* sequences from the top of the list. As a result, the selected sequences are more closely related to Protein X than the unselected ones. The number of unselected sequences is 80–10 × *i*. The sequence set with the larger index *i* contains all of the sequences with the smaller index. The MSA*i* is the MSA of the DATA*i*. In addition, we assume that the top 70 sequences have the same structures as that of Protein X, and their functional residues are located in exactly the same regions as in Protein X. In contrast to the 70 sequences, the remaining 10 sequences have the same structures as that of Protein X, but their functional regions are located in different regions from those in Protein X. The residues in the functional regions are shown by the red spheres on the structures depicted on the left side of Figure 2.

In MSA1, the alignment sites corresponding to the functional residues are highly conserved. Additionally, a certain number of residues in non-functional regions also have high conservation scores, due to the close evolutionary relationship to Protein X. Consequently, the unconserved residues in non-functional regions as well as the conserved residues in functional regions are surrounded by conserved residues. Then, the Moran scatterplot is drawn for MSA1, and is referred to as Moran’s scatterplot I. The regression line in the scatterplot has a gentle slope.

The slope of the regression line of Moran’s scatterplot II is expected to be slightly steeper than that of Moran’s scatterplot I when we consider MSA2, which includes 10 slightly more divergent sequences from Protein X. The assumption here is that a small number of residues within non-functional regions have been substituted with different ones, while almost all of the residues in the functional regions are still conserved in MSA2. Therefore, the distribution of the plots in Moran’s scatterplot II is expected to change slightly, since clusters of unconserved residues should be observed in the non-functional regions. Plots corresponding to residues with low conservation scores in the non-functional regions are expected to appear at the bottom-left in Moran’s scatterplot II. This means that a certain number of unconserved residues in the non-functional regions are surrounded by unconserved residues. Therefore, the DSPAC derived from Moran’s scatterplot II is considered to become slightly greater than that from Moran’s scatterplot I.

Likewise, in the Moran scatterplots III, IV, V, VI and VII, the slopes would gradually become steeper. When the MSA consists of the sequences that moderately diverged from Protein X, the fraction of unconserved residues in the non-functional regions increases. Simultaneously, the fraction of conserved residues in the non-functional regions decreases. On the other hand, the fraction of conserved residues in the functional regions either remains the same or slightly decreases. The difference in the decreasing rates of conserved residues between the functional and non-functional regions creates the contrast in the conservation scores between the functional and non-functional regions, and would lead to the intensification of the DSPAC. Therefore, if the MSAs are arranged in the ascending order of the DSPAC in the corresponding Moran scatterplots, then the order would be MSAs 1, 2, 3, 4, 5, 6 and 7.

However, MSA8 would be expected to show a different trend. As assumed above, MSA8 includes the sequences of DATA8, which are the most diverged from Protein X, and their functional residues are located on completely different regions from those in Protein X. Consequently, when these sequences are added to the MSA, the conservation scores of the sites corresponding to the functional residues of Protein X decrease. Therefore, the difference in the conservation scores between the functional and non-functional regions in MSA8 becomes smaller than that in MSA7. Then, ideally, the DSPAC of Moran’s scatterplot VIII becomes smaller than that of Moran’s scatterplot VII. As shown at the bottom of Figure 2, the plots of the DSPACs as a function of the order of the datasets are expected to show a convex shape. In this case, MSA7 provides the maximum DSPAC. Accordingly, the set of sequences corresponding to MSA7 is adopted as the most appropriate set of sequences for the functional region prediction of Protein X.

#### Details of the prediction procedure

**Figure 3:**
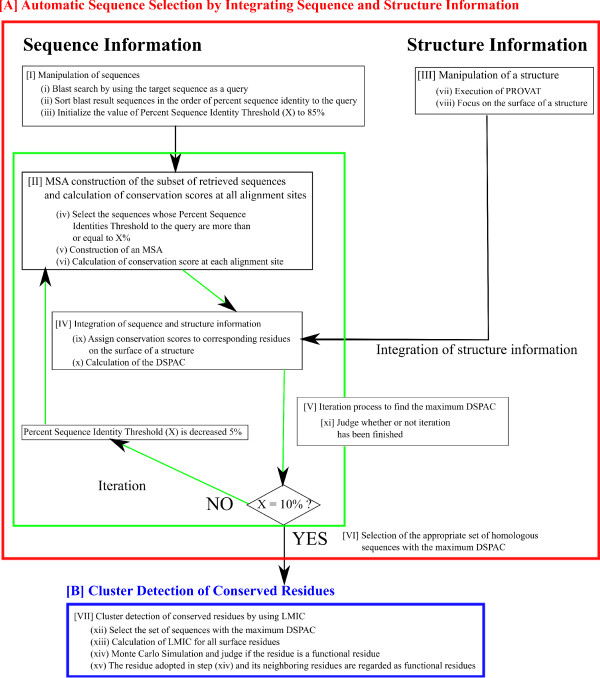
**Flow chart of our prediction method.** The method consists of seven steps and is roughly classified into two parts, [**A**] Appropriate Sequence Selection for Functional Region Prediction (I-VI) and [**B**] Cluster Detection of Conserved Residues (VII). [I] Manipulation of sequences, [II] Manipulation of a structure, [III] MSA construction of the subset of retrieved sequences and calculation of conservation scores at all alignment sites, [IV] Integration of sequence and structure information, [V] Iteration process to find the maximum DSPAC, [VI] Selection of the appropriate set of homologous sequences with the maximum DSPAC, [VII] Cluster detection of conserved residues by using LMIC.

As shown in Figure 3, our method is roughly divided into two parts, [A] Appropriate Sequence Selection for Functional Region Prediction and [B] Cluster Detection of Conserved Residues.

[A] Appropriate Sequence Selection for Functional Region Prediction

The sequence selection procedure is divided into two processes, the Data Preprocessing Process and the Iteration Process. The Data Preprocessing Process consists of two steps (Boxes [I] and [II]). The Iteration Process consists of four steps (Boxes [III], [IV], [V], and [VI]). The maximum DSPAC, which is expected to indicate the most appropriate set of sequences, was found by decreasing the threshold of the percent sequence identity to Protein X from 85% to 10%, with intervals of 5%. Hereafter, this threshold is referred to as the percent sequence identity threshold. The first and second parts of the prediction procedures (Boxes [I] and [II]) are preprocessing steps of the sequence data and the structure data, respectively, for the selection of an appropriate set of sequences. The third part of the procedure (Box [III]) is the MSA construction of the sequences. The information obtained from Box [II] is integrated with that from Box [III] in Box [IV], in which the calculated conservation scores are assigned to the corresponding residues on the query’s tertiary structure. In Box [IV], the existence of the spatial autocorrelation of the conservation score was examined on the structure. After the iteration, we adopted the set of sequences with the maximum DSPAC.

[I] *Manipulation of sequences*

The procedure described in this section corresponds to Box [I] in Figure 3. At first, we collected the homologous sequences of a target protein by a BLAST search [23] with an *e*-value parameter of 1*e* + 3. Among the retrieved sequences, the sequences with lengths that were shorter than 80% or longer than 150% of the length of the target sequence were removed.

[II] *Manipulation of a structure*

The procedure described in this section corresponds to Box [II] in Figure 3. As described above, the protein structure is represented as a residue-residue interaction network. Let’s consider a protein that forms a complex with another molecule, and define its inner, surface and interface residues for other molecules. Before executing PROVAT, all of the HETATM record lines in the PDB file were removed. During the PROVAT process, at first, a number of H_2_O atoms are scattered around, covering the target protein molecule. We set C_β_ as the representative atom for each residue except for Gly, for which C_α_ is the representative atom. C_β_ and H_2_O are considered to be the nodes to be tessellated. The Voronoi tessellation is then performed. A node in the tessellation is called a meta site. Hereafter, we focus on the meta sites, which correspond to the C_β_ atoms. The meta sites neighboring meta site *i* are called the slave sites of meta site *i*. If at least one H_2_O is included in the slave sites around meta site *i*, then the residue corresponding to meta site *i* is considered to be exposed on the surface. If at least one atom of the other protein molecule is included in the slave sites around meta site *i*, then the residue corresponding to meta site *i* is considered to be an interface residue to the other molecule. Otherwise, the residue corresponding to meta site *i* is regarded as an inner residue.

[III] *MSA construction of the subset of retrieved sequences and calculation of the conservation scores at all alignment sites*

All of the sequences retrieved by the BLAST search were aligned by using MAFFT (ver 6.239) [38]. The sequences with an identity to the query that was more than or equal to X% sequence identity were then adopted and aligned, again. The conservation score was calculated at each alignment site by the equation (*B*).

[IV] *Integration of the information from* [II] *and* [III]

The information obtained from the sequence and structure preprocessing is integrated at this step; that is, the conservation scores are assigned to the corresponding residues on the tertiary structure. During the iteration to evaluate the sequence sets, the information from step [III] is identical. Then, the DSPAC for the conservation scores is calculated, as an index reflecting the appropriateness of the set of sequences for functional region prediction. As mentioned above, a Moran scatterplot is often used to display the existence of spatial autocorrelation. In our method, the horizontal axis represents the conservation score of residue *i*, while the vertical axis is the average conservation score over the neighboring residues of residue *i*. The average conservation score over the neighboring residues around residue *i* is defined as follows:

(C) $$A_{i}=\frac{\sum_{j=1,j\neq i}^{N}w_{\text{ij}}\left(y_{j}-\bar{y}\right)}{n}$$

where *y*_*j*_ is the conservation score of residue *i*. $\bar{y}$ is the average conservation score of all surface residues. *w*_*ij*_ is the same as that used in equation (A). A neighboring residue around residue *j* is defined as the residue with a degree of connection on the Voronoi surface less than or equal to three and that simultaneously exists within a distance of *D*Å from the C_*β*_ of residue *i*. *D* is shifted from 5.0Å to 9.0Å, with intervals of 0.5Å. We used the slope of the regression line of a Moran scatterplot as the DSPAC. The correlation coefficient was calculated in each Moran scatterplot as the slope of the regression line. *n* is the number of neighboring residues of residue *i*.

[V] *Iteration process to find the maximum DSPAC*

As shown in Figure 3, procedures [III], [IV] and [V] are iterated while shifting the percent sequence identity threshold.

[VI] *Selection of the appropriate set of homologous sequences with the maximum DSPAC*

The DSPACs are plotted as a function of the percent sequence identity threshold to the target protein. Among the 16 conditions, we adopted the condition corresponding to the set of sequences with the maximum DSPAC. The percent sequence identity corresponding to the maximum DSPAC is referred to as the “adopted percent sequence identity”.

[B] Cluster Detection of Conserved Residues

[VII] Cluster detection of conserved residues by using Local Moran’s I of Conservation scores (LMIC)

We adopted the strategy to detect clusters of conserved residues on the molecular surface of a protein. Therefore, we designed a score to evaluate the degree of clustering of conserved residues around each residue by modifying Local Moran’s I [30,31], which is referred to as the Local Moran’s I by using Conservation score (LMIC). The LMIC of residue *i* (LMIC_*i*_) is defined as follows:

(D) $$LMIC_{i}=\frac{\left({\text{Cons}}_{i}-\bar{Cons}\right)\sum_{j=1,j\neq i}^{N}w_{\text{ij}}\left({\text{Cons}}_{j}-\bar{\text{Cons}}\right)}{\sigma^{2}}$$

where Cons_*i*_ is the conservation score of surface residue *i*. Cons_*j*_ is that of surface residue *j*. $\bar{\text{Cons}}$ is the average of the conservation scores of all surface residues. σ is the standard deviation of the conservation scores over all surface residues. *w*_*ij*_ is the same as that used in the equation (A), which is a weighting factor to define a neighboring relationship between residues *i* and *j*. Due to *w*_*ij*_, the conservation score of a spatially distant residue *j* from residue *i* does not contribute to LMIC_*i*_. The nodes that have similar conservation scores and are adjacent to each other yield a large LMIC_*i*_. A Monte Carlo simulation was performed to test the statistical significance of LMIC_*i*_ in the following procedure. Firstly, the coordinates and the conservation score of residue *i* were fixed. Secondly, the coordinates of the other surface residues were randomly exchanged with each other. These steps were iterated 10,000 times and finally, a total of 10,000 datasets, consisting of attributes and coordinates, were generated. If the original LMIC_*i*_ is larger than the lowest score of the top 5% of those of the generated 10,000 datasets, and simultaneously the conservation score of residue *i* is larger than the average of the conservation scores of all surface residues, then the residues around the conserved residue *i* are regarded as predicted functional residues.

### Evaluation method

#### Functional region information

In this work, the prediction targets of our method are the functional regions. These regions are expected to be suitable for our method, due to the accumulation of conserved residues at certain places on the surface. The catalytic residues listed in the Catalytic Site Atlas [24] and the residues within 9Å from the geometric center of the catalytic residues were regarded as constituents of the functional regions. The distance of 9Å was adopted, based on the observation by Chelliah *et al.*[39].

Hereafter, the term ‘functional region’ refers to both the actual catalytic residues and their surrounding residues that reside within 9Å from the geometric center of the catalytic residues, while ‘predicted functional region’ means the functional region predicted by our method.

#### Evaluation scores

The performance of the functional region prediction for each protein was evaluated with the following quantities:

(E) $$\begin{array}{lll} F-\text{score} & = & (2\times \text{Sensitivity}\times \text{Specificity})/(\text{Sensitivity}\\ & & +\text{Specificity})\\ \text{Sensitivity} & = & TP/(TP+FN)\\ \text{Specificity} & = & TP/(TP+FP)\\ \text{Selectivity} & = & TN/(TN+FP) \end{array}$$

Among them, we mainly used the F-score, which is a measure that combines and harmonizes sensitivity and specificity, to evaluate the functional region prediction performance of our method. This is because it is inappropriate to determine which method shows the best performance among various datasets by using either the sensitivity or specificity. In general, when the sensitivity increases, the specificity decreases and vice versa. Hence, the sensitivities, specificities and selectivities are also shown in Additional file 1: Figure SA, Additional file 2: Figure SB, Additional file 3: Figure SC, respectively, as Additional information.

## Results

Our functional region prediction method consists of two steps, the selection of appropriate homologous sequences and the detection of conserved residue clusters on a structure. We used DSPAC for the appropriate sequence selection, and LMIC for the cluster detection. Hence, we evaluated the performance of the DSPAC-based sequence selection method (DSPAC-based method) and that of the cluster detection by LMIC.

Firstly, in order to evaluate the performance of the DSPAC-based method, we compared the average F-score of the predictions with the set of sequences collected by the DSPAC-based method and those of two other sequence selection methods without structure information, Naïve approaches and SDPfox [40]. Then, LMIC was used for the cluster detection of conserved residues. As described in the Methods, the set of sequences selected by the DSPAC-based method was the set with the maximum DSPAC, among the subsets of homologous sequences generated by the procedure described in the Methods. SDPfox was originally developed to divide an MSA into some functional sub-groups and to identify the specificity-determining positions in each sub-group [40]. We used SDPfox because the sequences classified into the same sub-group as that of the target protein would be suitable for the functional region prediction of the query, since the proteins in the sub-group are supposed to share their functional regions. Therefore, the SDPfox-based sequences are the sequences in one of the sub-groups automatically identified by SDPfox, which contains the target protein. The sets of sequences identified by Naïve approaches are the 16 different sets of sequences constructed during the DSPAC-based sequence selection. Each set contains the sequences with percent sequence identities that are more than or equal to the specified percent sequence identity threshold to the target protein sequence. The percent sequence identity threshold was shifted from 85% to 10%, with intervals of 5%. The sequence set with the smaller threshold contains all of the sequences with larger thresholds.

Secondly, in order to evaluate the performance of LMIC as a detection method for conserved residue clusters, we compared the performances of the predictions with LMIC and those with PatchFinder. PatchFinder finds the patch of residues with the lowest probability of occurring by chance [10,11]. At first, PatchFinder sets a minimal average conservation cutoff for a patch, to generate the biggest patch with an average conservation score that is higher than or equal to the cutoff. In order to generate a conserved patch, each of the 10 residues with the highest conservation score on the protein surface is selected as a starting point. In the extension stage, the most highly conserved residue among the neighboring residues is added to the existing patch, and the average conservation score of the new patch is calculated. Then, a neighboring residue is defined as the residue with at least one heavy atom within 4Å from at least one atom of a residue under consideration. If the average conservation score of the patch is higher than the cutoff, then the new residue is accepted and the extension stage is repeated. The search procedure ends when the average conservation score of the patch drops below the cutoff. This procedure is repeated while shifting the cutoff, and the same numbers of patches and cutoffs are generated. Finally, the patch with the maximum likelihood is chosen.

**Figure 4:**
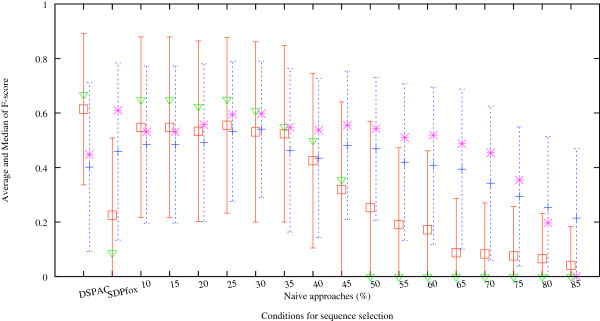
**The average and median F-scores of the functional region predictions generated by 18 different criteria for sequence selection.** The horizontal axis represents the sequence selection methods, DSPAC-based method, SDPfox, and Naïve approaches, with 16 different percent sequence identity thresholds. The red open rectangles with error bars indicate the averaged F-scores of the predictions when LMIC was used to detect the cluster of conserved residues. The negative part of the error bar, which would extend into the negative F-score region, is not drawn, since the F-value has a positive value by definition. The light green open triangles indicate the medians of the predictions when LMIC was used to detect the cluster of conserved residues. The blue crosses with error bars indicate the averaged F-scores of the predictions when PatchFinder was used to detect the cluster of conserved residues. The purple asterisks indicate the medians of the predictions when PatchFinder was used to detect the cluster of conserved residues. The combination of DSPAC-based sequence selection and LMIC showed the best performance among all combinations of the methods. The prediction performances by the PatchFinder and Naïve approaches at 30% sequence identity are comparable to those by the LMIC and DSPAC-based approaches.

In Figure 4, the averaged F-scores of the functional region predictions over the 54 proteins under consideration are shown. The neighboring residue threshold for each protein was fixed at the value corresponding to the maximum DSPAC. The red open rectangles with error bars indicate the averaged F-scores of the predictions when LMIC was used to detect the cluster of conserved residues. The light green open triangles indicate the medians of the predictions when LMIC was used to detect the cluster of conserved residues. We compared the performance of the DSPAC-based method with those of the SDPfox and Naïve approaches. The DSPAC-based method showed the maximum average F-score among the three methods. The difference between the average F-score obtained by the DSPAC-based method and those by the SDPfox and Naïve approaches with the threshold of more than 35% sequence identity was statistically significant (*p* < 0.01) by the paired *t*-test. The differences between the F-scores by the DSPAC-based method and those by the Naïve approaches with the threshold of less than or equal to 35% sequence identity were statistically significant (*p* < 0.05) by the paired *t*-test. These observations indicate that the DSPAC-based method has improved the accuracies of functional region predictions. Roughly speaking, the plots of the average F-scores by Naïve approaches form a convex shape. The average F-scores by Naïve approaches with the threshold values ranging from 85% to 25% monotonously increase. The maximum average F-score is obtained by the Naïve approach with the 25% sequence identity threshold. The average F-score generated by SDPfox is smaller than those obtained by the other methods, except for the Naïve approaches with the threshold of more than 55% sequence identity.

Next, we show the performance of cluster detection with PatchFinder. The blue crosses indicate the averaged F-scores of functional region predictions over the 54 proteins under consideration, when PatchFinder was used for the detection of conserved residue clusters. The purple asterisks indicate the medians of the predictions when PatchFinder was used to detect the cluster of conserved residues. The average F-score obtained by the DSPAC-based method is smaller than the F-scores generated by the Naïve and SDPfox approaches, except for the Naïve approaches with thresholds of more than 60% sequence identity. Roughly speaking, the plots of the average F-scores by Naïve approaches form a convex shape. The average F-scores by Naïve approaches with the threshold values ranging from 85% to 30% monotonously increase. The maximum average F-score is obtained by the Naïve approach with the threshold of 30% sequence identity. The differences between the F-score by Naïve approaches with the threshold of 30% sequence identity with PatchFinder (0.531) and that by the DSPAC-based method with LMIC (0.614) were not statistically significant by the paired *t*-test. The average F-scores generated by Naïve approaches with the threshold values ranging from 25% to 10% decrease. These observations indicate that the prediction performances by the PatchFinder and Naïve approaches at 30% sequence identity are comparable to those by the LMIC and DSPAC-based approaches.

We compared the average F-score obtained with LMIC (red rectangles) to that generated with PatchFinder (blue crosses), using the identical sequence set. The average F-score from the DSPAC-based method with LMIC (0.614) is larger than that from PatchFinder (0.402), and is statistically significant by the paired *t*-test (*p* < 0.01). When we used the set of sequences selected by the Naïve approaches with thresholds ranging from 40% to 10%, the average F-scores with LMIC (0.547, 0.547, 0.533, 0.555, 0.531, 0.524, 0.425) were larger than those with PatchFinder (0.484, 0.484, 0.492, 0.533, 0.541, 0.463, 0.435), although the average F-score with LMIC (0.531) is slightly smaller than that with PatchFinder at the threshold of 30% identity (0.541). In contrast, the average F-scores with LMIC (0.319, 0.253, 0.191, 0.172, 0.087, 0.083, 0.075, 0.065, 0.041) were smaller than those with PatchFinder (0.481, 0.469, 0.420, 0.407, 0.394, 0.343, 0.294, 0.254, 0.215), when we used the set of sequences selected by the Naïve approaches with the thresholds ranging from 45% to 80%. In the same way, the average F-scores determined with LMIC (0.225) were smaller than those obtained with PatchFinder (0.459), when we used the set of sequences selected by SDPfox.

### Two application examples

**Figure 5:**
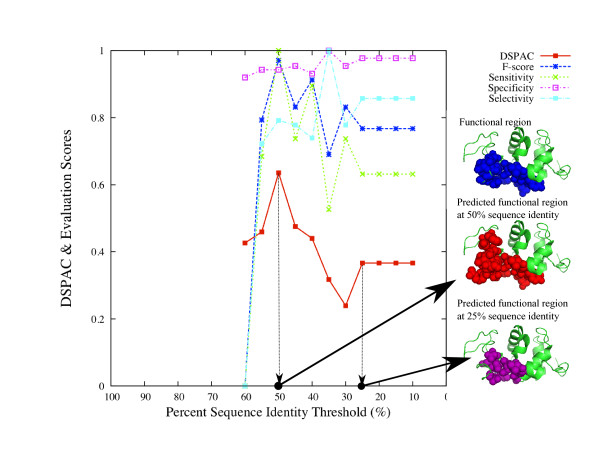
**Variations of the DSPAC and evaluation scores of the analysis of C-type lysozyme.** Each score is plotted as a function of the percent sequence identity threshold. The real functional residues, the functional residues predicted by using the set of sequences with the maximum DSPAC, and the functional residues predicted by using the set of sequences at the 25% sequence identity threshold are shown by blue, red and purple spheres on the C-type lysozyme structures, respectively.

**Figure 6:**
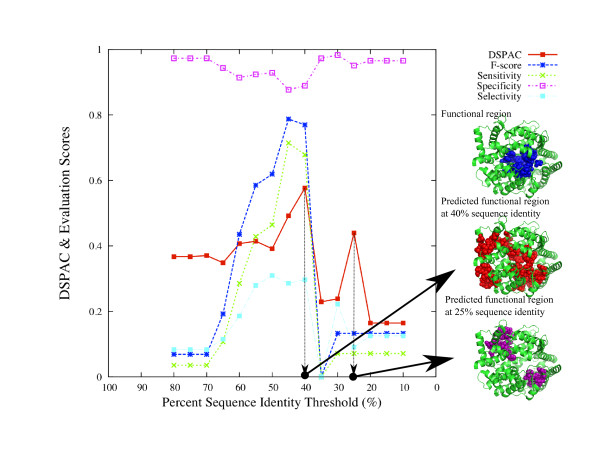
**Variation of the DSPAC and evaluation scores of the analysis of ACE2.** Each score is plotted as a function of the percent sequence identity threshold. The real functional residues, the functional residues predicted by using the set of sequences with the maximum DSPAC, and the functional residues predicted by using the set of sequences at the 25% sequence identity threshold are shown by blue, red and purple spheres on the ACE2 structures, respectively.

Here, we show the analyses of two proteins, canine C-type lysozyme (1el1 chain A) and human angiotensin converting enzyme 2 (ACE2) (1o8a chain A) in Figures 5 and 6, respectively, as examples of the application of the combined DSPAC and LMIC approach to the functional region prediction. These proteins were chosen because the homologues of the two proteins include representative cases of functional divergence, as mentioned in the Discussion.

#### Example 1: C-type lysozyme

The DSPAC, F-score, sensitivity, specificity and selectivity for the prediction of C-type lysozyme are plotted as a function of the percent sequence identity threshold (Figure 5), by closed squares with a solid line, asterisks with a dashed line, crosses with a dotted line, open squares with a dotted-dashed line and closed squares with a dotted-dashed line, respectively. The plot is drawn when the neighboring residue threshold is 7.0Å, because the maximum DSPAC is obtained when the threshold to define neighboring residues is 7.0Å. Then, the DSPAC plots form a convex shape. Likewise, the F-score, sensitivity and selectivity show similar patterns. The maximum DSPAC (0.64) is observed at the 50% sequence identity threshold, which corresponds to the percent sequence identity threshold with the maximum F-score (0.97) and sensitivity (1.0). In contrast, the specificity is almost flat, although the lowest score is observed at the 60% sequence identity threshold. The maximum score of selectivity (1.00) is observed at the 35% sequence identity threshold, although both the F-score (0.69) and sensitivity (0.53) are low, as compared to the percent sequence identity threshold with the maximum DSPAC. The DSPAC and the scores corresponding to 70, 75, 80, and 85% are not plotted, because the numbers of sequences in the datasets at these thresholds are less than 10. In this way, the set of sequences with the maximum DSPAC would provide a high F-score and better sensitivity.

On the right side of the plot, there are three small windows in tandem, where C-type lysozyme structures are shown. In the top window, 19 real functional residues are represented by blue spheres. In the middle window, 24 functional residues, predicted by using the set of sequences with the maximum DSPAC, are represented by red spheres. Among them, 19 residues corresponded to the real functional residues, while five predicted residues did not. In the bottom window, 18 residues, predicted by using the set of sequences at a 25% sequence identity threshold, are represented by purple spheres. The 25% sequence identity threshold was selected for comparison because the average F-score for the 54 proteins is the maximum among the Naïve approaches. All of the residues corresponded to the real functional residues. These observations also showed the high performance of the DSPAC-based sequence selection. However, the coverage of the predicted residues against the real functional residues was lower than that in the case of the maximum DSPAC.

#### *Example 2:* Angiotensin converting enzyme 2 (*ACE2)*

Next, we describe the analysis of ACE2. In the same way as the prediction for lysozyme, the DSPAC, F-score, sensitivity, specificity and selectivity for the prediction of ACE2 are plotted as a function of the percent sequence identity threshold (Figure 6), by closed squares with a solid line, asterisks with a dashed line, crosses with a dotted line, open squares with a dotted-dashed line and closed squares with a dotted-dashed line, respectively. The plot is drawn when the neighboring residue threshold is 7.0Å. The DSPAC, F-score, sensitivity and selectivity show similar patterns, although the specificity (open squares with a dotted-dashed line) is almost flat. The percent sequence identity threshold for the maximum DSPAC (40%) and those for the F-score and sensitivity (45%) are close to each other. The score corresponding to 85% is not plotted, because the numbers of sequences in the datasets at these thresholds are less than 10.

As in the case of the application to the C-type lysozyme described above, there are three small windows in tandem, where ACE2 structures are shown. In the top window, 28 real functional residues are represented by blue spheres. In the middle window, 64 functional residues, predicted by using the set of sequences with the maximum DSPAC, are represented by red spheres. Among them, 19 residues corresponded to the real functional residues. However, the functional meanings of the remaining 45 predicted residues have not been characterized. In the bottom window, 26 residues, predicted by using the set of sequences at a 25% sequence identity threshold, are represented by purple spheres. No residue corresponded to the real functional residues. In the same manner as that for C-type lysozyme, the DSPAC-based sequence selection showed high performance for the appropriate sequence selection.

## Discussion

We evaluated the performance of the DSPAC-based method and that of the cluster detection by LMIC. At first, we will discuss the differences in the performance of functional region prediction among the three sequence selection methods, under the conditions where LMIC was used to detect clusters of conserved residues. The plots of the average F-scores for Naïve approaches formed a convex shape, with the maximum average F-score at the threshold with 25% sequence identity. This reminds us of the percent sequence identities limit reported by Todd *et al.*[15], which reserves the first three digits of the EC numbers for enzymes conserved within the same family. That is, more than 30% or 40% sequence identity would be useful as an empirical criterion to select homologous sequences for functional region prediction. However, our study suggested that it is better to determine the criterion of the sequence selection for each protein, instead of using a fixed threshold, because some proteins share the same functions with closely related homologues, while others share the same functions with diverged ones and closely related ones. Hence, we compared the sequence selection efficiency among Naïve approaches, SDPfox and DSPAC. Contrary to our expectation, the average F-score by SDPfox, 0.225, was smaller than those of the other methods, except for the Naïve approaches with thresholds ranging from 55% to 85% (0.191, 0.172, 0.087, 0.083, 0.075, 0.065, 0.041). Consider the minimum percent sequence identity between the sequences in the optimum sequence set for functional region prediction selected by SDPfox and the target sequence. The average minimum percent sequence identity over the target sequences examined in this study was 62.6%. The average F-scores of Naïve approaches with the 60% and 65% sequence sets were 0.172 and 0.087, respectively. These are significantly smaller than the maximum average F-score generated by the Naïve approach with 25% sequence identity. Therefore, the sets of sequences selected by SDPfox tend to consist of less divergent sequences, as compared to the sets obtained by the Naïve approach with 25% sequence identity. This might occur because SDPfox was originally developed to discriminate between the ligand- or substrate-binding specificities among homologous proteins. In contrast to the average F-score obtained by SDPfox, the average F-score by the DSPAC-based sequences was the maximum among the average F-scores by all sequence selection methods. Thus, the sequence selection by the DSPAC-based method improved the performance of functional region prediction. The distribution of the adopted percent sequence identity thresholds of the DSPAC-based method is shown in Additional file 4: Figure SD in the Additional information. The average of the adopted percent sequence identity thresholds for all target proteins was 27.1%, which was close to the percent sequence identity threshold corresponding to the maximum average F-score by Naïve approaches, 25%. Therefore, it is better to construct the set of sequences for functional region prediction for each protein by using DSPAC, instead of a fixed threshold.

Next, we will discuss the differences in the performance of cluster detection of conserved residues between LMIC and PatchFinder. Roughly speaking, the plots of the average F-scores against the percent sequence identity form convex shapes, regardless of the cluster detection method. The maximum average F-scores are obtained by the Naïve approaches with similar thresholds for LMIC (25%) and PatchFinder (30%), although the maximum score with LMIC is larger than that with PatchFinder. The prediction performances of both LMIC and PatchFinder monotonously decline by the Naïve approaches with the percent identity thresholds less than 25%. As shown in Figure 4, the decreasing rate of the average F-scores determined by Naïve approaches from 25% to 10% with PatchFinder is larger than that with LMIC. The average F-scores generated with LMIC were larger than those with PatchFinder when we used the Naïve approaches with thresholds smaller than 40% sequence identity, except for 30% sequence identity. Likewise, the average F-scores obtained with LMIC were larger than those with PatchFinder when we used the DSPAC-based sequence selection. In contrast, the average F-scores obtained with PatchFinder were larger than those with LMIC when we used the Naïve approaches with 30% and larger than 35% sequence identities. Likewise, the average F-scores generated with PatchFinder were larger than those with LMIC when we used SDPfox. The average of the minimum percent sequence identities of the sequences with the queries in the SDPfox-based sets is 62.6%. Thus, LMIC is considered to suit more divergent sequences than PatchFinder for functional region prediction. Therefore, LMIC should be used to detect clusters of conserved residues, when a set of sequences is constructed and includes at least one sharing less than 40% sequence identity with the target protein.

Accordingly, cluster detection with LMIC by using the DSPAC-based sequences is considered to provide the best performance. In fact, the combination of DSPAC-based sequence selection and LMIC showed the best performance among all combinations of the methods. In the next section, we will show two results of the analyses, as examples.

### Examples: Exclusion of functionally diverged homologues from the functional region prediction

We discuss the details of the analyses of two proteins, canine C-type lysozyme (Figure 5) and human ACE2 (Figure 6), as examples of the application of the combined DSPAC and LMIC approach.

As described in the Introduction, the non-enzyme proteins, the α-lactalbumins, belong to the same family as the enzymes, C-type lysozymes. In the analysis shown in Figure 5, no α-lactalbumin sequence was included in any sets of sequences, in which the percent sequence identity threshold from the canine C-type lysozyme sequence was greater than or equal to 50%. The maximum DSPAC was observed at 50% sequence identity. The decline of the DSPAC was observed at the 45% sequence identity threshold. The set of sequences corresponding to the threshold of less than 50% includes the α-lactalbumin sequences. Thus, the DSPAC was successfully able to distinguish the sequence set including only C-type lysozyme from that including both C-type lysozyme and α-lactalbumin. When the set of sequences including α-lactalbumin was used for the prediction, the prediction accuracy was lower than that with the set of sequences with the maximum DSPAC.

In the analysis of ACE2, the maximum DSPAC was observed at 40% sequence identity. The declines of the DSPAC and F-score for the prediction of ACE2 were observed at a 35% sequence identity threshold (Figure 6). These declines coincide with the inclusion of the ACE2 homologue with different substrate specificity, dipeptidyl carboxypeptidase, in the sequence set. No dipeptidyl carboxypeptidase sequence was included in any sets of sequences, in which the percent sequence identity threshold from the human ACE2 sequence was larger than or equal to 40%. Accordingly, the DSPAC was successfully able to distinguish the set including only ACE2 sequences from that including both ACE2 and dipeptidyl carboxypeptidase. Human ACE2 and its homologue, *E. coli* dipeptidyl carboxypeptidase (1y79 chain 1), share the cavity for the catalytic reaction at the corresponding structural position. However, the catalytic sites of ACE2 (1o8a A: 353, 354, 384, 513, 523, 1y79 A: 498, 614) reside in different locations in the cavity from those of the dipeptidyl carboxypeptidase, and the locations of the catalytic sites do not overlap.

#### Comparison with other methods

As described in the Introduction, there are two other methods to address the problem of selecting appropriate homologous sequences for functional region prediction. However, it was difficult to compare the performances of the methods, since the methods used to evaluate the performances are different not only from each other, but also from the method adopted in this study. Here, we describe the similarities and differences among the methods.

In the method reported by Aloy *et al*. [8], the prediction is made based on the iterative evaluation of the clustering of the conserved residues on the tertiary structure. In this meaning, their approach is similar to ours. The major difference between their method and ours resides in the selection of the sequence set for the functional region prediction. In their method, the initial MSA consists of divergent sequences. If no spatial clustering of conserved residues is identified, then the distant homologues to the target protein are removed. The construction of the MSA and the identification of conserved residues are iterated until the cluster of conserved residues is identified. In contrast, our method constructs 18 sequence sets with different percent sequence identity thresholds. The DSPACs of all the sets are calculated, and the set with the maximum DSPAC is selected for the functional region prediction.

Mihalek *et al.*[12,17] used an index called the “residue clustering measure” to evaluate the appropriateness of an MSA for functional surface prediction. The purpose of their work was to select an appropriate subset of sequences from a curated set of homologous sequences in the HSSP (homology-derived secondary structure of proteins) database [41]. At first, each residue is ranked with the rvET score in ascending order of the residue importance [12,17]. After the residues are ranked, they calculated a value for a selection function, *S*_*c*_, which assigns 1 if residue *i* belongs to the top fraction *c* of all residues in the protein. Using *S*_*c*_, they defined the clustering weight, *w*_*c*_. This score is designed to have a large value when the number of conserved residues, which are not adjacent in the primary structure but are close in the tertiary structure, is large. Then, the integral of the *z*-score of *w*_*c*_ over the sequence length is calculated. Finally, *A*_*clustering*_ is calculated by dividing the integral by the sequence length. The sequence sets generated by selecting various nodes on a phylogenetic tree, based on the Metropolis Monte Carlo simulation, are evaluated by using *A*_*clustering*_ as the optimization score. Our approach is different from their method [12,17], although both strategies consider the importance of the structural information of proteins as a guide to select the sequences for an MSA. In Mihalek’s method, the set of sequences is generated by the Monte Carlo method. In our method, only 18 sequence sets were generated, by shifting the percent sequence identity threshold. This could be the advantage of our method, as compared to Mihalek’s method. However, the percent sequence identity is not always a good index to estimate the functional differences. Therefore, even if the DSPAC is adequate for the evaluation of the set of sequences, the appropriate set might not be included in the candidate sets. The possible improvements and extensions of our method regarding this disadvantage will be described in the Concluding remarks. As described above, Mihalek *et al*. utilized the rvET-based score, *A*_*clustering*_, which is calculated by iterating the *w*_*c*_ calculation while shifting the fraction *c*. In contrast, our method used DSPAC, which is simply calculated as the correlation coefficient of a Moran scatterplot about the conservation scores. This could be another advantage of our method. These two advantages would make it possible to search through the vast sequence space quickly. Our method would be especially useful with enormous amounts of sequence information.

## Conclusions

We have developed a novel index to select the sequences appropriate for the evaluation of residue conservation, and implemented the index within our method to predict the functional regions of a protein. The implementation of the index improved the performance of the functional region prediction. The index represents the degree of clustering of conserved residues on the tertiary structure of the protein. For this purpose, structure and sequence information were integrated within the index by the application of spatial statistics. The benefits and the pitfalls of the new method were discussed, based on the results of the applications. Finally, we would like to conclude by describing possible improvements and extensions of our method.

As described in the Introduction, Mihalek *et al.*[12,17] used an index called the “residue clustering measure” to evaluate the appropriateness of an MSA for functional surface prediction. We addressed a similar problem by a different approach. However, we could not compare the performance of our method and that of their method, since they did not report any evaluation scores used in this study, and their method is not available through the Internet. Hence, we will extend our method by introducing the strength of their method. As mentioned in the Methods, in our current study, the appropriate sequence set is selected from some candidate sequence sets. Each candidate is constructed by collecting the sequences with percent sequence identities from the query that are larger than or equal to a certain threshold. In contrast, in the method by Mihalek *et al*., a candidate sequence set is constructed by combining some sequence clusters on a phylogenetic tree of the target protein and its homologues. The construction of candidate sets by considering the topology of a phylogenetic tree could improve the performance of the appropriate sequence selection by the DSPAC-based sequence selection.

The second point is the extension of DSPAC to the template selection in homology modeling, by the application of the DSPAC-based sequence selection. The main purpose of structure modeling is to investigate molecular function. In recent years, the number of structures in the PDB has grown rapidly [42], which increases the chance of enhancing the accuracy of homology-based modeling by selecting the proper template structure. Homology modeling is applied to a sequence when its crystal structure is not available yet. It would be better to use the structure of a protein with a function considered to be the same as or similar to the amino acid sequence under consideration as a template. However, the structures retrieved by sequence similarity search and fold recognition programs do not always have the same or similar function as that of the target sequence, because such programs do not directly evaluate the functional similarity between the target sequence and a certain structure. Here, we consider the inverse problem. Suppose that we have a structure. The problem is to determine which homologous sequences can be modeled with the given structure as the template. A simple sequence similarity search may not provide an answer to the problem, since the functional similarity to the structure-known protein is not considered in the sequence similarity search. The DSPAC-based approach can be used to solve the problem, since the sequence set constructed by the maximum DSPAC criterion shares the same or similar biochemical functions.

The third point is the application of DSPAC-based sequence selection to the proteins with other types of functions. In this work, the performance of our method was evaluated by the application to monomeric enzymes. Therefore, we cannot tell if DSPAC-based sequence selection works for the proteins with other types of functions, for example, protein-protein interaction interface, *etc*. However, in fact, Mihalek *et al*. demonstrated that the performance of their method for protein-protein interaction interfaces was lower than that for active sites. The same might be true for our method. In such a case, we will improve our method to analyze proteins with different types of functions.

Spatial statistics has been used in population biology, in fields such as ecology and epidemiology. This work is the first application of spatial statistics to molecular biology. In this study, we applied the method to protein structure. The applicability of spatial statistics is not restricted to the protein structure data. The techniques of spatial statistics would be useful for the analyses of other molecular and cellular systems with spatial structures. Spatial statistics will thus be a promising tool for various fields in bioinformatics.

## Abbreviations

MSA: Multiple sequence alignment; DSPAC: Degree of spatial autocorrelation; LMIC: Local Moran’s I by using Conservation score; PDB: Protein Data Bank; ACE2: Angiotensin converting enzyme 2; rASA: Relative accessible surface area.

## Competing interests

The author(s) declare that they have no competing interests.

## Authors' contributions

WN contributed to the experimental design, and the data analysis and interpretation. HT contributed to the data interpretation. Both WN and HT wrote the manuscript, and gave final approval of the version to be submitted. All authors read and approved the final manuscript.

## Supplementary Material

Additional file 1 **Figure SA.** The average and median sensitivities of the functional region predictions generated by 18 different criteria for sequence selection. The horizontal axis represents the sequence selection methods, DSPAC-based method, SDPfox, and Naïve approaches, with 16 different percent sequence identity thresholds. The red open rectangles with error bars indicate the averaged sensitivities of the predictions when LMIC was used to detect the cluster of conserved residues. The negative part of the error bar, which would extend into the region of negative sensitivity, is not drawn, since the sensitivity has a positive value by definition. The light green open triangles indicate the medians of the predictions when LMIC was used to detect the cluster of conserved residues. The blue crosses with error bars indicate the averaged sensitivities of the predictions when PatchFinder was used to detect the cluster of conserved residues. The purple asterisks indicate the median sensitivities of the predictions when PatchFinder was used to detect the cluster of conserved residues. Click here for file

Additional file 2 **Figure SB.** The average and median specificities of the functional region predictions generated by 18 different criteria for sequence selection. The horizontal axis represents the sequence selection methods, DSPAC-based method, SDPfox, and Naïve approaches, with 16 different percent sequence identity thresholds. The red open rectangles with error bars indicate the averaged specificities of the predictions when LMIC was used to detect the cluster of conserved residues. The negative part of the error bar, which would extend into the region of negative specificity, is not drawn, since the specificity has a positive value by definition. The light green open triangles indicate the medians of the predictions when LMIC was used to detect the cluster of conserved residues. The blue crosses with error bars indicate the averaged specificities of the predictions when PatchFinder was used to detect the cluster of conserved residues. The purple asterisks indicate the median specificities of the predictions when PatchFinder was used to detect the cluster of conserved residues. Click here for file

Additional file 3 **Figure SC.** The average and median selectivities of the functional region predictions generated by 18 different criteria for sequence selection. The horizontal axis represents the sequence selection methods, DSPAC-based method, SDPfox, and Naïve approaches, with 16 different percent sequence identity thresholds. The red open rectangles with error bars indicate the averaged selectivities of the predictions when LMIC was used to detect the cluster of conserved residues. The negative part of the error bar, which would extend into the region of negative selectivity, is not drawn, since the selectivity has a positive value by definition. The light green open triangles indicate the medians of the predictions when LMIC was used to detect the cluster of conserved residues. The blue crosses with error bars indicate the averaged selectivities of the predictions when PatchFinder was used to detect the cluster of conserved residues. The purple asterisks indicate the median selectivities of the predictions when PatchFinder was used to detect the cluster of conserved residues. Click here for file

Additional file 4 **Figure SD.** The distribution of the adopted percent sequence identity thresholds by DSPAC-based sequence selection. The horizontal axis represents the percent sequence identity threshold. The vertical axis represents the percentage of the target proteins with the maximum DSPAC adopted at the percent sequence identity threshold. Click here for file
