# Supplementary figures and images for: Functional region prediction with a set of appropriate homologous sequences-an index for sequence selection by integrating structure and sequence information with spatial statistics
Source: BMC Struct Biol. 2012 May 29;12:11. doi: 10.1186/1472-6807-12-11 (PMC3533907; doi:10.1186/1472-6807-12-11)

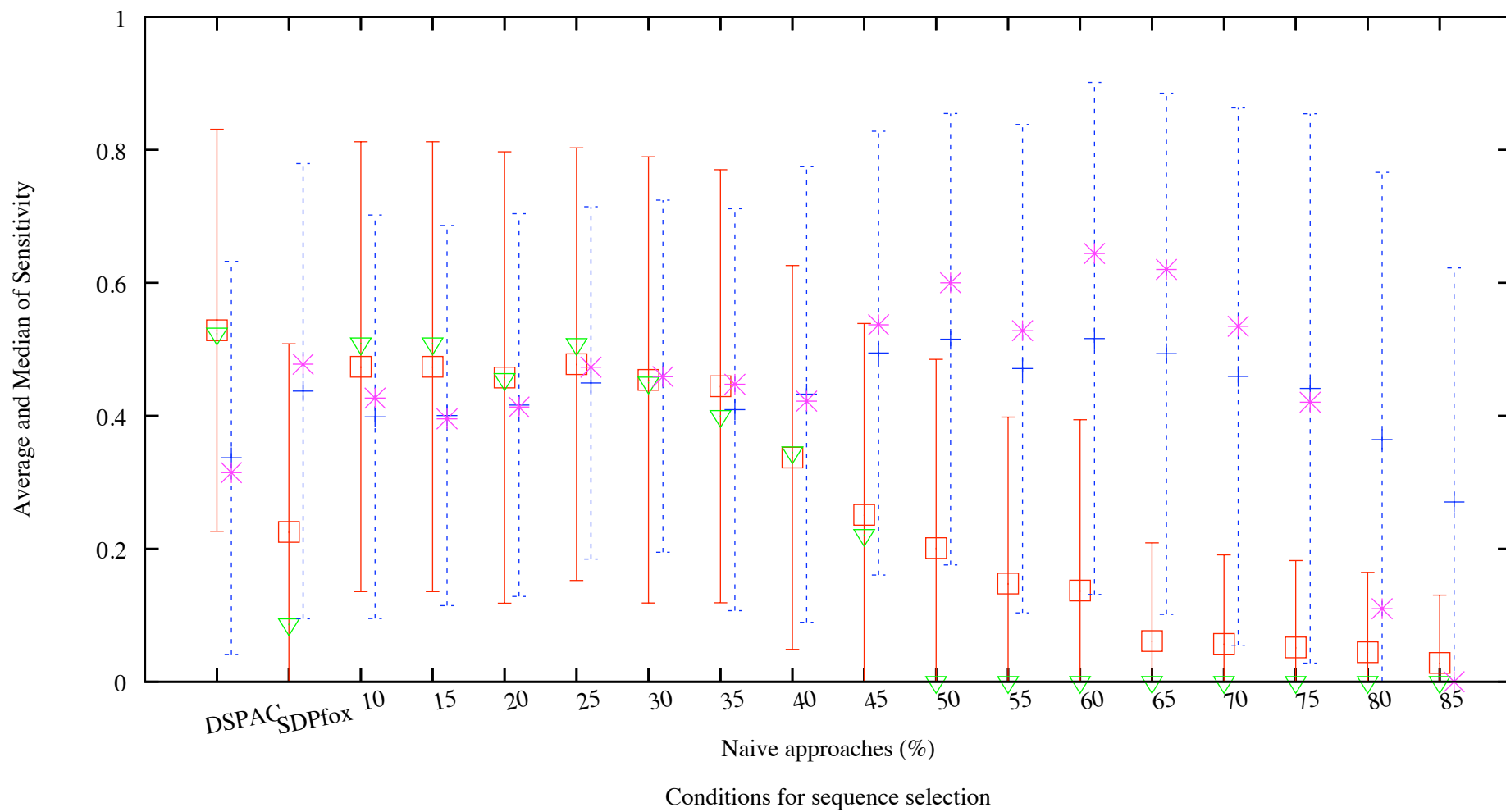

Supplement: Additional file 1 — Figure SA. The average and median sensitivities of the functional region predictions generated by 18 different criteria for sequence selection. The horizontal axis represents the sequence selection methods, DSPAC-based method, SDPfox, and Naïve approaches, with 16 different percent sequence identity thresholds. The red open rectangles with error bars indicate the averaged sensitivities of the predictions when LMIC was used to detect the cluster of conserved residues. The negative part of the error bar, which would extend into the region of negative sensitivity, is not drawn, since the sensitivity has a positive value by definition. The light green open triangles indicate the medians of the predictions when LMIC was used to detect the cluster of conserved residues. The blue crosses with error bars indicate the averaged sensitivities of the predictions when PatchFinder was used to detect the cluster of conserved residues. The purple asterisks indicate the median sensitivities of the predictions when PatchFinder was used to detect the cluster of conserved residues. [file 1472-6807-12-11-S1.pdf]

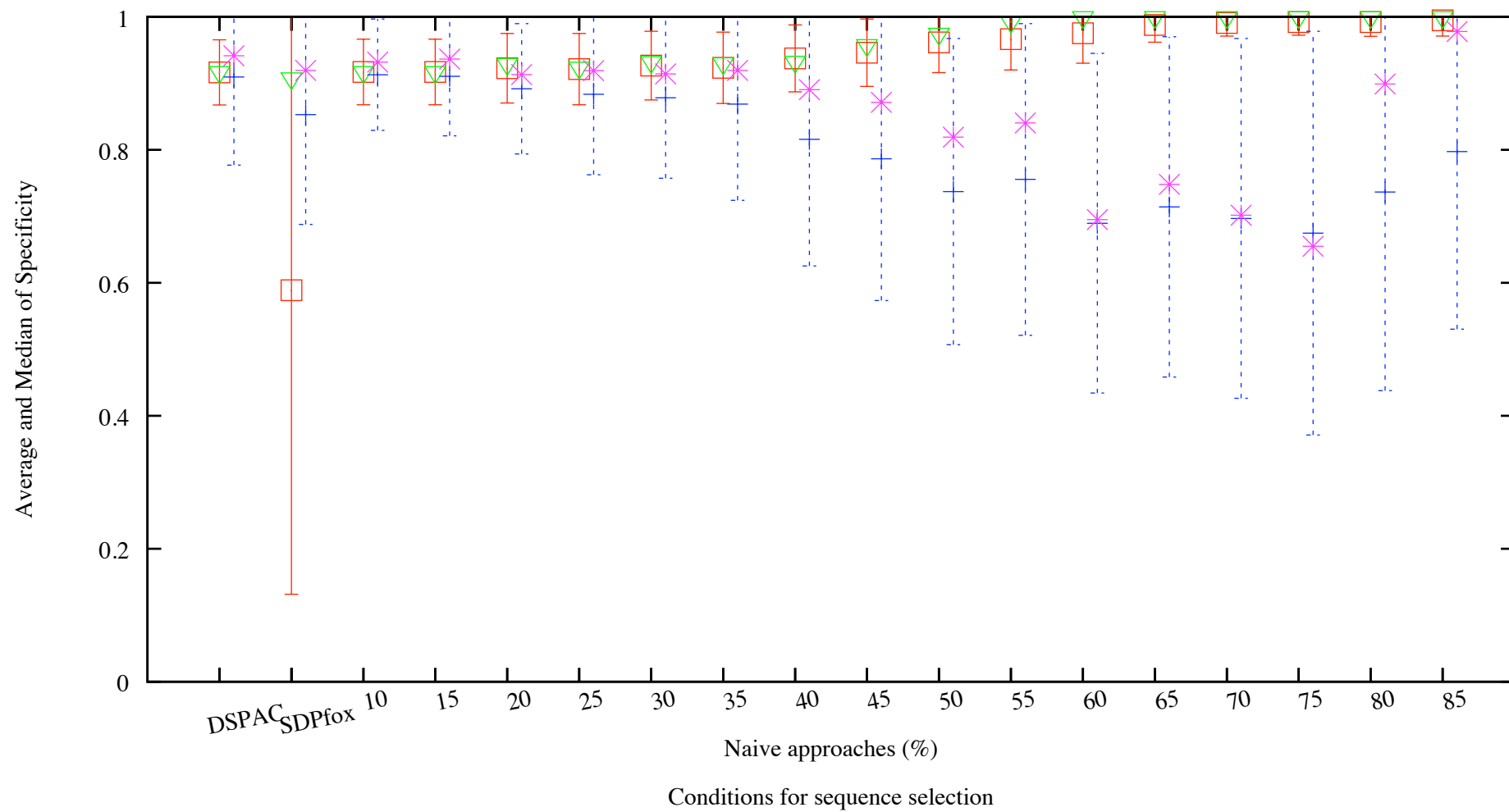

Supplement: Additional file 2 — Figure SB. The average and median specificities of the functional region predictions generated by 18 different criteria for sequence selection. The horizontal axis represents the sequence selection methods, DSPAC-based method, SDPfox, and Naïve approaches, with 16 different percent sequence identity thresholds. The red open rectangles with error bars indicate the averaged specificities of the predictions when LMIC was used to detect the cluster of conserved residues. The negative part of the error bar, which would extend into the region of negative specificity, is not drawn, since the specificity has a positive value by definition. The light green open triangles indicate the medians of the predictions when LMIC was used to detect the cluster of conserved residues. The blue crosses with error bars indicate the averaged specificities of the predictions when PatchFinder was used to detect the cluster of conserved residues. The purple asterisks indicate the median specificities of the predictions when PatchFinder was used to detect the cluster of conserved residues. [file 1472-6807-12-11-S2.pdf]

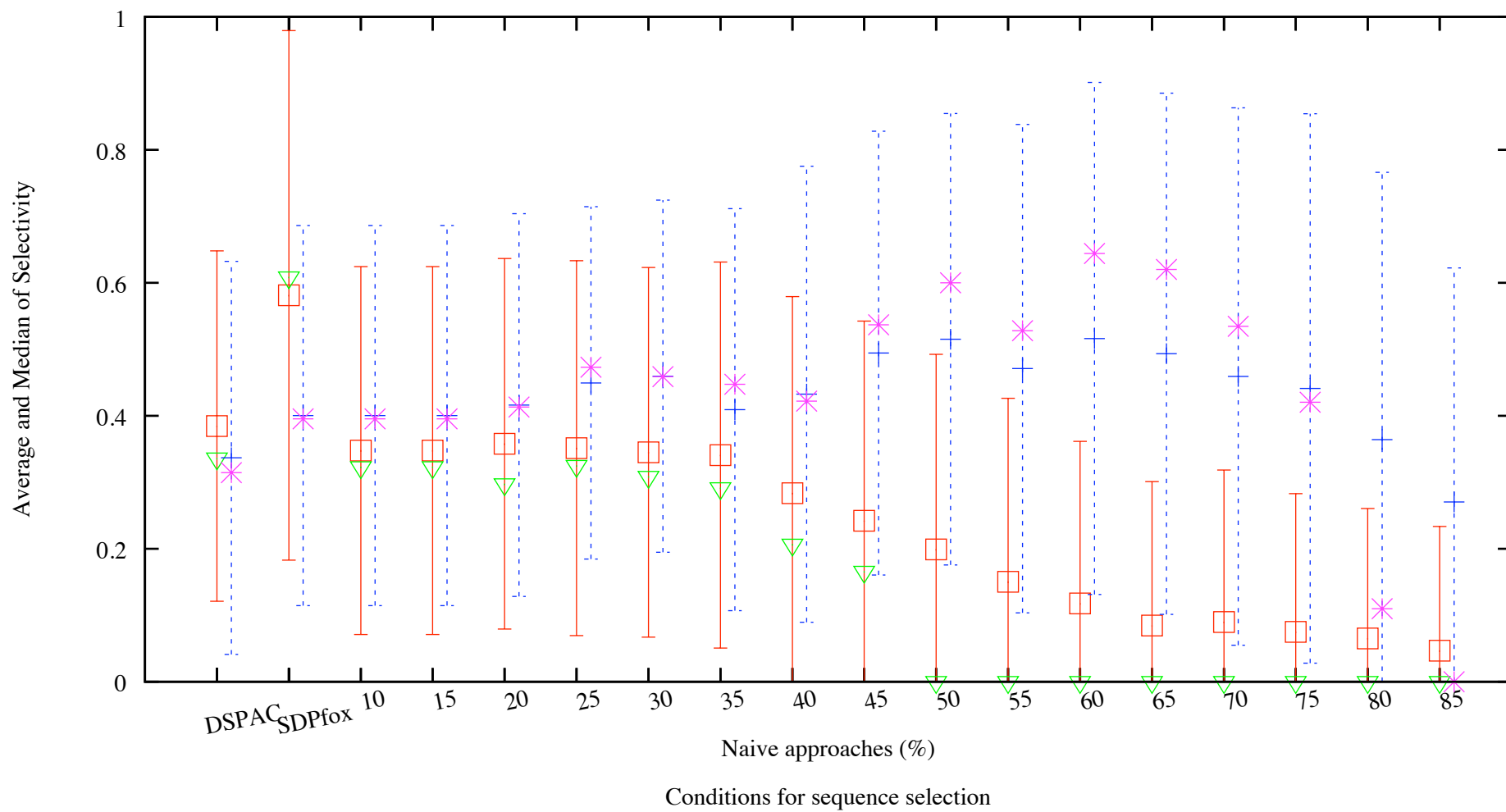

Supplement: Additional file 3 — Figure SC. The average and median selectivities of the functional region predictions generated by 18 different criteria for sequence selection. The horizontal axis represents the sequence selection methods, DSPAC-based method, SDPfox, and Naïve approaches, with 16 different percent sequence identity thresholds. The red open rectangles with error bars indicate the averaged selectivities of the predictions when LMIC was used to detect the cluster of conserved residues. The negative part of the error bar, which would extend into the region of negative selectivity, is not drawn, since the selectivity has a positive value by definition. The light green open triangles indicate the medians of the predictions when LMIC was used to detect the cluster of conserved residues. The blue crosses with error bars indicate the averaged selectivities of the predictions when PatchFinder was used to detect the cluster of conserved residues. The purple asterisks indicate the median selectivities of the predictions when PatchFinder was used to detect the cluster of conserved residues. [file 1472-6807-12-11-S3.pdf]

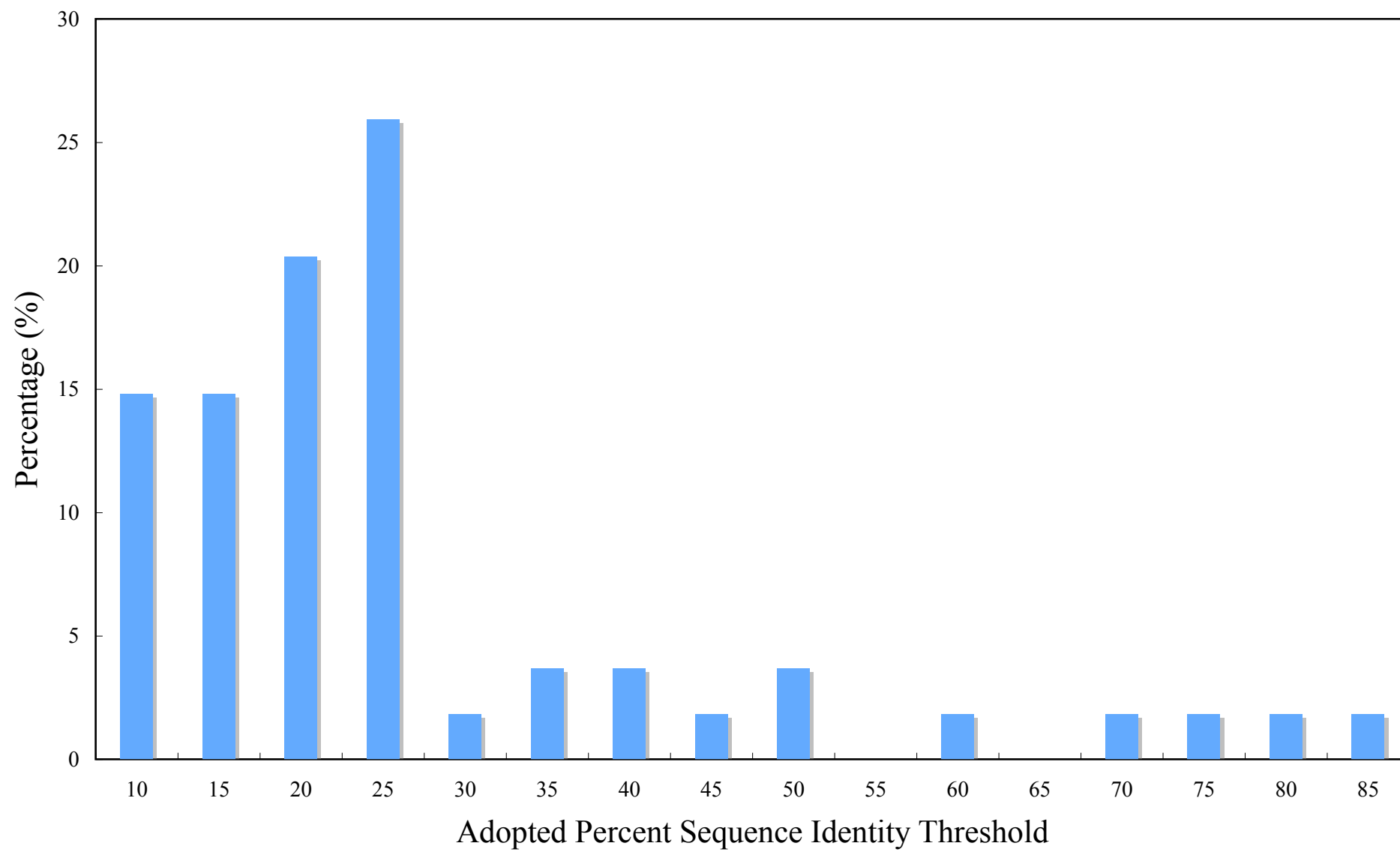

Supplement: Additional file 4 — Figure SD. The distribution of the adopted percent sequence identity thresholds by DSPAC-based sequence selection. The horizontal axis represents the percent sequence identity threshold. The vertical axis represents the percentage of the target proteins with the maximum DSPAC adopted at the percent sequence identity threshold. [file 1472-6807-12-11-S4.pdf]
